# 4-(Di­methyl­amino)­pyridinium trichlorido[4-(di­methyl­amino)­pyridine-κ*N*]cobaltate(II)

**DOI:** 10.1107/S1600536813015602

**Published:** 2013-06-12

**Authors:** Fatiha Guenifa, Nasreddine Hadjadj, Ouahida Zeghouan, Lamia Bendjeddou, Hocine Merazig

**Affiliations:** a Unité de Recherche Chimie de l’Environnement et Moléculaire Structurale, ’CHEMS’, Faculté des Sciences Exactes, Campus Chaabet Ersas, Université Constantine I, 25000 Constantine, Algeria

## Abstract

In the anion of the title compound, (C_7_H_11_N_2_)[CoCl_3_(C_7_H_10_N_2_)], the Co^II^ ion is coordinated by one N atom from a 4-(di­methyl­amino)­pyridine (DMAP) ligand and three Cl atoms, forming a CoNCl_3_ polyhedron with a distorted tetra­hedral geometry. In the crystal, cations and anions are linked *via* weak N—H⋯Cl and C—H⋯Cl hydrogen bonds. Double layers of complex anions stack along the *b*- axis direction, which alternate with double layers of 4-(di­methyl­amino)-pyridinium cations.

## Related literature

For applications and properties of DMAP, see: Araki *et al.* (2005 ▶); Satgé *et al.* (2004 ▶). For Co—N and Co—Cl bond lengths and angles in related compounds, see: Akbarzadeh Torbati *et al.* (2010 ▶); Baker *et al.* (1988 ▶). For hysrogen-bond motifs, see: Bernstein *et al.* (1995 ▶);
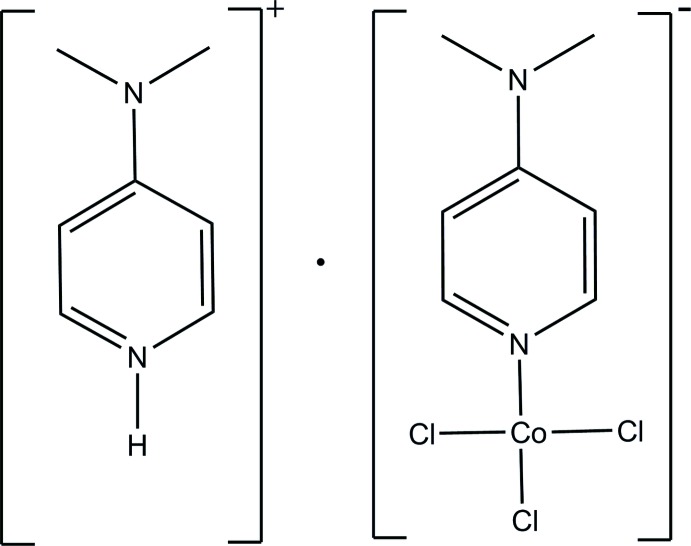


## Experimental

### 

#### Crystal data

(C_7_H_11_N_2_)[CoCl_3_(C_7_H_10_N_2_)]
*M* *_r_* = 410.63
Triclinic, 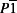

*a* = 7.7468 (2) Å
*b* = 8.4036 (2) Å
*c* = 15.4765 (4) Å
α = 79.732 (2)°
β = 89.983 (2)°
γ = 67.889 (2)°
*V* = 916.02 (4) Å^3^
*Z* = 2
Mo *K*α radiation
μ = 1.38 mm^−1^
*T* = 293 K
0.1 × 0.09 × 0.08 mm

#### Data collection

Bruker APEXII diffractometer
7932 measured reflections
3230 independent reflections
2982 reflections with *I* > 2σ(*I*)
*R*_int_ = 0.012

#### Refinement

*R*[*F* ^2^ > 2σ(*F* ^2^)] = 0.021
*wR*(*F* ^2^) = 0.054
*S* = 1.04
3230 reflections
199 parameters
H-atom parameters constrained
Δρ_max_ = 0.31 e Å^−3^
Δρ_min_ = −0.24 e Å^−3^

### 

Data collection: *APEX2* (Bruker, 2006 ▶); cell refinement: *SAINT* (Bruker, 2006 ▶); data reduction: *SAINT*; program(s) used to solve structure: *SIR2002* (Burla *et al.*, 2005 ▶); program(s) used to refine structure: *SHELXL97* (Sheldrick, 2008 ▶); molecular graphics: *ORTEP-3 for Windows* (Farrugia, 2012 ▶); software used to prepare material for publication: *WinGX* (Farrugia, 2012 ▶), *Mercury* (Macrae *et al.*, 2006 ▶) and *POVRay* (Persistence of Vision Team, 2004 ▶).

## Supplementary Material

Crystal structure: contains datablock(s) global, I. DOI: 10.1107/S1600536813015602/vm2195sup1.cif

Structure factors: contains datablock(s) I. DOI: 10.1107/S1600536813015602/vm2195Isup2.hkl

Additional supplementary materials:  crystallographic information; 3D view; checkCIF report

## Figures and Tables

**Table 1:** Selected bond lengths (Å)

Co—Cl1	2.2482 (6)
Co—Cl2	2.2642 (5)
Co—Cl3	2.2680 (5)
Co—N2	2.0154 (14)

**Table 2:** Hydrogen-bond geometry (Å, °)

*D*—H⋯*A*	*D*—H	H⋯*A*	*D*⋯*A*	*D*—H⋯*A*
N4—H4⋯Cl2^i^	0.86	2.64	3.3535 (17)	142
N4—H4⋯Cl3^i^	0.86	2.70	3.3279 (17)	131
C13—H13⋯Cl3^i^	0.93	2.81	3.4048 (19)	123

Symmetry code: (i) 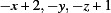.

**Fig. 1.**
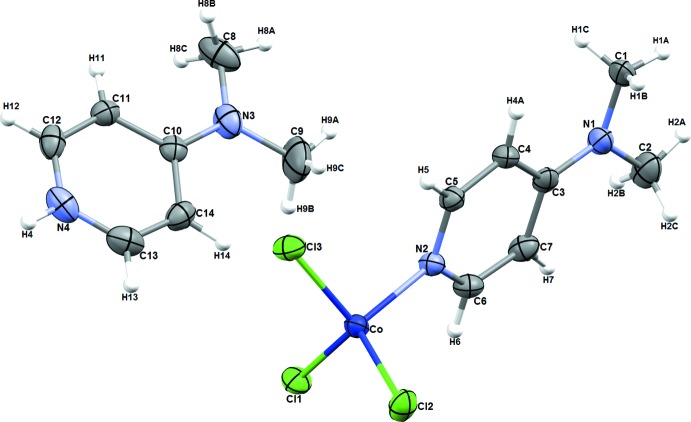
The asymmetric unit of the title compound with the atom numbering scheme. Displacement ellipsoids are drawn at the 50% probability level and H atoms are represented as spheres of arbitrary radii.

**Fig. 2.**
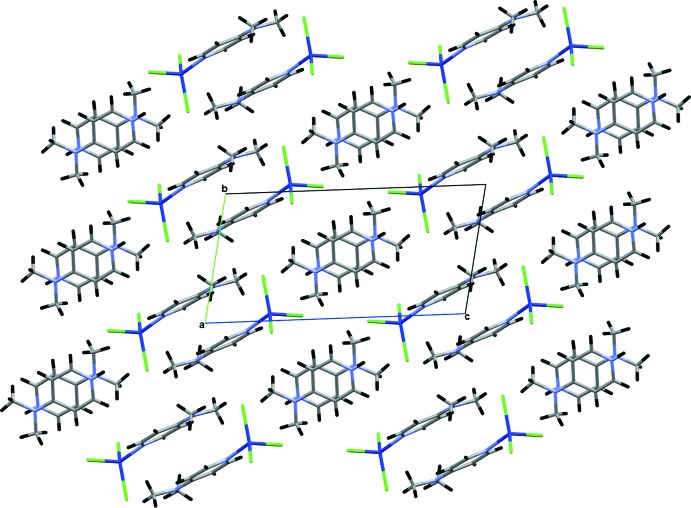
View of the crystal structure of (I), showing the alternating double layers of cations and anions along the b axis.

**Fig. 3.**
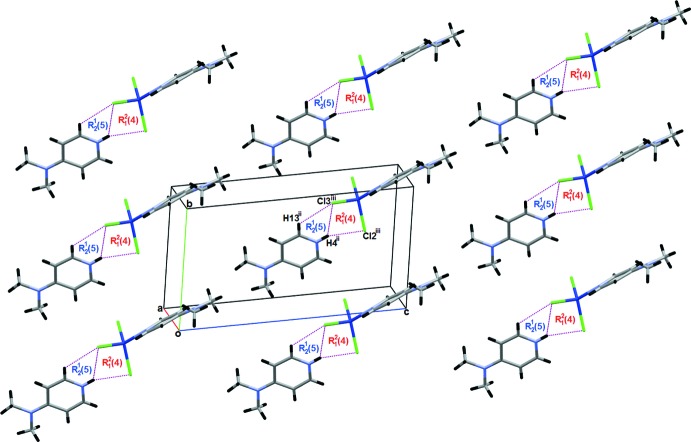
Part of the crystal structure, showing the aggregation of R12(5) and R21 (4) hydrogen-bonding motifs. [Symmetry codes: (ii) 1-x, 1-y, 1-z; (iii) -1+x, 1+y, z]

## supplementary crystallographic information

### Comment

The N-heteroaromatic ligand 4-(dimethylamino) pyridine (DMAP) finds use as a homogeneous catalyst in cellulose acylation in the synthesis of biodegradable plastics (Satgé *et al.*, 2004). DMAP is also known to form transition metal complexes which exhibit luminescence properties (Araki *et al.*, 2005). We report here the synthesis and crystal structure of such a cobalt(II) complex with 4-(dimethylamino)pyridine.

The title compound (I) consists of one complex anion [CoCl_3_(C_7_H_10_N_2_)]^-^ and one 4-(dimethylamino)-pyridinium cation (Figure 1). In the structure of (I), each cobalt(II) is coordinated by one N atom from the DMAP ligand and three Cl atoms, forming a distorted tetrahedral coordination geometry. The Co—N and Co—Cl bond lengths and angles (Table 1) are within normal range as observed in: dichloro(6,6'-dimethyl-2,2'-bipyridyl)cobalt(II) hemibenzene solvate (Baker *et al.*, 1988) and dichlorido(6,6'-dimethyl-2, 2'-bipyridine-κ^2^N,N')cobalt(II) (Akbarzadeh Torbati *et al.*, 2010) .

The crystal structure of the title compound (I) is formed by double layers of complex anions [CoCl_3_(C_7_H_10_N_2_)]^-^ stacking along the b axis, at *c* = 0 and 1, which alternate with double layers of 4-(dimethylamino)-pyridinium cations placed along the [010] direction at *c* = 1/2 (Figure 2). The crystal packing is consolidated by two N—H···Cl and one C—H···Cl hydrogen bonds established between cations and anions, forming rings in two-dimensional network which can be described by the graph-set motif R^1^_2_(5) and R^2^_1_ (4) (Bernstein *et al.*, 1995) (Figure 3).

### Experimental

A mixture of NaN_3_ and CoCl_2_.6H_2_O in methanol was stirred for half an hour, then 4-dimethylaminopyridine was added to the solution and the reaction continued to stir for one hour. After filtration, the pink filtrate was allowed to stand at room temperature. Blue crystals were obtained by slow evaporation.

### Refinement

The H atoms were placed at calculated positions with C—H = 0.93 and 0.96 Å, for aromatic and methyl H atoms, respectively, with *U*_iso_(H) = 1.2*U*_eq_(C) for aromatic H atoms and 1.5*U*_eq_(C) for methyl H atoms.

### Crystal data

**Table:**

(C_7_H_11_N_2_)[CoCl_3_(C_7_H_10_N_2_)]	*Z* = 2
*M**_r_* = 410.63	*F*(000) = 422
Triclinic, *P*1	*D*_x_ = 1.489 Mg m^−^^3^
Hall symbol: -P 1	Mo *K*α radiation, λ = 0.71073 Å
*a* = 7.7468 (2) Å	Cell parameters from 3230 reflections
*b* = 8.4036 (2) Å	θ = 2.7–25.0°
*c* = 15.4765 (4) Å	µ = 1.38 mm^−^^1^
α = 79.732 (2)°	*T* = 293 K
β = 89.983 (2)°	Prism, blue
γ = 67.889 (2)°	0.1 × 0.09 × 0.08 mm
*V* = 916.02 (4) Å^3^	

### Data collection

**Table:**

Bruker APEXII diffractometer	2982 reflections with *I* > 2σ(*I*)
Radiation source: fine-focus sealed tube	*R*_int_ = 0.012
Graphite monochromator	θ_max_ = 25.0°, θ_min_ = 2.7°
φ scans	*h* = −9→9
7932 measured reflections	*k* = −9→9
3230 independent reflections	*l* = −18→18

### Refinement

**Table:**

Refinement on *F*^2^	0 restraints
Least-squares matrix: full	H-atom parameters constrained
*R*[*F*^2^ > 2σ(*F*^2^)] = 0.021	*w* = 1/[σ^2^(*F*_o_^2^) + (0.0266*P*)^2^ + 0.4298*P*] where *P* = (*F*_o_^2^ + 2*F*_c_^2^)/3
*wR*(*F*^2^) = 0.054	(Δ/σ)_max_ = 0.002
*S* = 1.04	Δρ_max_ = 0.31 e Å^−^^3^
3230 reflections	Δρ_min_ = −0.24 e Å^−^^3^
199 parameters	

### Special details

**Table:**

Geometry. Bond distances, angles etc. have been calculated using the rounded fractional coordinates. All su's are estimated from the variances of the (full) variance-covariance matrix. The cell esds are taken into account in the estimation of distances, angles and torsion angles
Refinement. Refinement of *F*^2^ against ALL reflections. The weighted *R*-factor *wR* and goodness of fit *S* are based on *F*^2^, conventional *R*-factors *R* are based on *F*, with *F* set to zero for negative *F*^2^. The threshold expression of *F*^2^ > σ(*F*^2^) is used only for calculating *R*-factors(gt) *etc*. and is not relevant to the choice of reflections for refinement. *R*-factors based on *F*^2^ are statistically about twice as large as those based on *F*, and *R*- factors based on ALL data will be even larger.

### Fractional atomic coordinates and isotropic or equivalent isotropic displacement parameters (Å^2^)

**Table:**

	*x*	*y*	*z*	*U*_iso_*/*U*_eq_	
Co	0.97567 (3)	−0.05454 (3)	0.75509 (1)	0.0213 (1)	
Cl1	1.17207 (6)	0.08718 (6)	0.73814 (3)	0.0306 (1)	
Cl2	1.12085 (6)	−0.34664 (5)	0.80434 (3)	0.0341 (1)	
Cl3	0.80521 (6)	−0.03086 (6)	0.63092 (3)	0.0309 (1)	
N1	0.4028 (2)	0.28626 (19)	1.01267 (9)	0.0296 (4)	
N2	0.78837 (18)	0.06985 (17)	0.83537 (9)	0.0226 (4)	
C1	0.2121 (2)	0.2955 (3)	1.00244 (12)	0.0344 (5)	
C2	0.4620 (3)	0.3238 (3)	1.09356 (12)	0.0404 (6)	
C3	0.5268 (2)	0.2194 (2)	0.95499 (10)	0.0240 (5)	
C4	0.4763 (2)	0.1638 (2)	0.88186 (10)	0.0247 (5)	
C5	0.6070 (2)	0.0912 (2)	0.82633 (10)	0.0235 (5)	
C6	0.8363 (2)	0.1278 (2)	0.90386 (11)	0.0261 (5)	
C7	0.7166 (2)	0.1992 (2)	0.96373 (11)	0.0279 (5)	
N3	0.5958 (2)	0.6338 (2)	0.60387 (10)	0.0348 (5)	
N4	0.8714 (2)	0.4027 (2)	0.40501 (11)	0.0345 (5)	
C8	0.4455 (3)	0.8064 (3)	0.58671 (16)	0.0464 (7)	
C9	0.6493 (4)	0.5499 (4)	0.69593 (13)	0.0620 (9)	
C10	0.6871 (2)	0.5610 (2)	0.53913 (11)	0.0242 (5)	
C11	0.6388 (2)	0.6428 (2)	0.44901 (11)	0.0261 (5)	
C12	0.7323 (3)	0.5605 (3)	0.38523 (11)	0.0318 (6)	
C13	0.9237 (3)	0.3224 (2)	0.48886 (14)	0.0359 (6)	
C14	0.8384 (2)	0.3958 (2)	0.55592 (12)	0.0309 (5)	
H1A	0.14222	0.34572	1.04879	0.0515*	
H1B	0.21388	0.17984	1.00525	0.0515*	
H1C	0.15458	0.36701	0.94653	0.0515*	
H2A	0.35548	0.37006	1.12652	0.0605*	
H2B	0.51870	0.40810	1.07920	0.0605*	
H2C	0.55093	0.21800	1.12820	0.0605*	
H4A	0.35311	0.17666	0.87136	0.0296*	
H5	0.56902	0.05397	0.77929	0.0281*	
H6	0.95919	0.11843	0.91068	0.0313*	
H7	0.75922	0.23461	1.01020	0.0334*	
H4	0.92757	0.35268	0.36341	0.0414*	
H8A	0.39712	0.83607	0.64133	0.0696*	
H8B	0.34751	0.80547	0.54908	0.0696*	
H8C	0.49274	0.89144	0.55842	0.0696*	
H9A	0.56759	0.62160	0.73266	0.0929*	
H9B	0.77569	0.53523	0.71003	0.0929*	
H9C	0.63979	0.43749	0.70578	0.0929*	
H11	0.54315	0.75273	0.43378	0.0313*	
H12	0.69889	0.61485	0.32650	0.0382*	
H13	1.02129	0.21331	0.50122	0.0430*	
H14	0.87896	0.33776	0.61367	0.0371*	

### Atomic displacement parameters (Å^2^)

**Table:**

	*U*^11^	*U*^22^	*U*^33^	*U*^12^	*U*^13^	*U*^23^
Co	0.0182 (1)	0.0204 (1)	0.0238 (1)	−0.0059 (1)	0.0025 (1)	−0.0039 (1)
Cl1	0.0251 (2)	0.0346 (2)	0.0351 (2)	−0.0161 (2)	0.0011 (2)	−0.0038 (2)
Cl2	0.0423 (3)	0.0219 (2)	0.0288 (2)	−0.0039 (2)	0.0040 (2)	−0.0005 (2)
Cl3	0.0260 (2)	0.0317 (2)	0.0315 (2)	−0.0057 (2)	−0.0046 (2)	−0.0092 (2)
N1	0.0294 (8)	0.0294 (8)	0.0233 (7)	−0.0030 (6)	0.0043 (6)	−0.0069 (6)
N2	0.0194 (7)	0.0225 (7)	0.0245 (7)	−0.0061 (5)	0.0014 (5)	−0.0054 (5)
C1	0.0273 (9)	0.0329 (10)	0.0304 (9)	0.0003 (7)	0.0098 (7)	−0.0014 (8)
C2	0.0501 (12)	0.0379 (11)	0.0266 (9)	−0.0065 (9)	0.0054 (8)	−0.0130 (8)
C3	0.0259 (8)	0.0168 (8)	0.0229 (8)	−0.0021 (6)	0.0010 (7)	−0.0015 (6)
C4	0.0187 (8)	0.0278 (9)	0.0251 (8)	−0.0068 (7)	0.0005 (6)	−0.0037 (7)
C5	0.0225 (8)	0.0251 (8)	0.0223 (8)	−0.0084 (7)	−0.0003 (6)	−0.0050 (7)
C6	0.0207 (8)	0.0256 (9)	0.0312 (9)	−0.0080 (7)	−0.0019 (7)	−0.0055 (7)
C7	0.0289 (9)	0.0257 (9)	0.0279 (9)	−0.0081 (7)	−0.0038 (7)	−0.0077 (7)
N3	0.0380 (9)	0.0459 (9)	0.0282 (8)	−0.0206 (7)	0.0062 (7)	−0.0161 (7)
N4	0.0364 (8)	0.0359 (9)	0.0430 (9)	−0.0214 (7)	0.0158 (7)	−0.0202 (7)
C8	0.0357 (11)	0.0520 (13)	0.0636 (14)	−0.0192 (10)	0.0152 (10)	−0.0358 (11)
C9	0.0711 (17)	0.097 (2)	0.0253 (10)	−0.0384 (15)	0.0099 (10)	−0.0160 (12)
C10	0.0245 (8)	0.0268 (9)	0.0269 (8)	−0.0157 (7)	0.0010 (7)	−0.0063 (7)
C11	0.0248 (8)	0.0235 (8)	0.0301 (9)	−0.0107 (7)	−0.0021 (7)	−0.0022 (7)
C12	0.0394 (10)	0.0409 (11)	0.0248 (9)	−0.0269 (9)	0.0027 (8)	−0.0047 (8)
C13	0.0287 (9)	0.0245 (9)	0.0568 (12)	−0.0121 (7)	0.0048 (9)	−0.0093 (9)
C14	0.0323 (9)	0.0274 (9)	0.0330 (9)	−0.0153 (8)	−0.0053 (8)	0.0026 (7)

### Geometric parameters (Å, º)

**Table:**

Co—Cl1	2.2482 (6)	C2—H2C	0.9600
Co—Cl2	2.2642 (5)	C2—H2A	0.9600
Co—Cl3	2.2680 (5)	C2—H2B	0.9600
Co—N2	2.0154 (14)	C4—H4A	0.9300
N1—C1	1.457 (2)	C5—H5	0.9300
N1—C2	1.459 (2)	C6—H6	0.9300
N1—C3	1.344 (2)	C7—H7	0.9300
N2—C5	1.352 (2)	C10—C11	1.422 (2)
N2—C6	1.349 (2)	C10—C14	1.420 (2)
N3—C9	1.456 (3)	C11—C12	1.358 (3)
N3—C10	1.331 (2)	C13—C14	1.347 (3)
N3—C8	1.457 (3)	C8—H8A	0.9600
N4—C12	1.339 (3)	C8—H8B	0.9600
N4—C13	1.336 (3)	C8—H8C	0.9600
N4—H4	0.8600	C9—H9A	0.9600
C3—C7	1.419 (2)	C9—H9B	0.9600
C3—C4	1.407 (2)	C9—H9C	0.9600
C4—C5	1.363 (2)	C11—H11	0.9300
C6—C7	1.363 (2)	C12—H12	0.9300
C1—H1B	0.9600	C13—H13	0.9300
C1—H1A	0.9600	C14—H14	0.9300
C1—H1C	0.9600		
			
Co···H4^i^	3.2300	C13···H8B^viii^	2.8000
Cl1···N2	3.3714 (16)	C14···H9B	2.8000
Cl1···C4^ii^	3.5458 (17)	C14···H9C	2.7800
Cl1···C5^ii^	3.6498 (17)	H1A···H2A	2.1400
Cl1···C6	3.6194 (17)	H1A···Cl2^vii^	3.0500
Cl1···C12^iii^	3.549 (2)	H1B···C7^vii^	2.9700
Cl2···N4^i^	3.3535 (17)	H1B···H4A	2.3400
Cl2···Cl3	3.5783 (7)	H1B···C4	2.7700
Cl3···N2	3.4083 (14)	H1B···C6^vii^	2.8800
Cl3···Cl2	3.5783 (7)	H1B···N2^vii^	2.9400
Cl3···C8^iv^	3.648 (3)	H1B···C5^vii^	3.0700
Cl3···C13^i^	3.4048 (19)	H1C···Cl2^xii^	2.8900
Cl3···N4^i^	3.3279 (17)	H1C···C4	2.7400
Cl3···C5	3.5318 (16)	H1C···H4A	2.2600
Cl1···H5^ii^	3.0400	H2A···H1A	2.1400
Cl1···H14	2.8700	H2A···C9^xi^	2.9400
Cl1···H12^iii^	3.0400	H2A···H9A^xi^	2.2800
Cl1···H4A^ii^	2.8500	H2B···H7	2.2900
Cl1···H8A^v^	2.8400	H2B···C7	2.7800
Cl1···H6	3.1400	H2B···C3^xi^	2.9600
Cl1···H2C^vi^	3.0700	H2B···C7^xi^	3.0600
Cl2···H1C^v^	2.8900	H2B···Cl2^vi^	3.1500
Cl2···H1A^vii^	3.0500	H2C···Cl1^vi^	3.0700
Cl2···H2B^vi^	3.1500	H2C···C7	2.8500
Cl2···H4^i^	2.6400	H2C···H7	2.4600
Cl3···H8C^iv^	3.0000	H4···Cl2^i^	2.6400
Cl3···H5	2.9500	H4···Co^i^	3.2300
Cl3···H4^i^	2.7000	H4···Cl3^i^	2.7000
Cl3···H8B^viii^	3.0300	H4A···Cl1^ix^	2.8500
Cl3···H11^viii^	2.8600	H4A···H1C	2.2600
Cl3···H13^i^	2.8100	H4A···H1B	2.3400
N2···Cl1	3.3714 (16)	H4A···C1	2.4900
N2···Cl3	3.4083 (14)	H5···Cl1^ix^	3.0400
N4···C14^iii^	3.394 (2)	H5···Cl3	2.9500
N4···Cl2^i^	3.3535 (17)	H6···Cl1	3.1400
N4···Cl3^i^	3.3279 (17)	H7···C2	2.5600
N2···H1B^vii^	2.9400	H7···H2B	2.2900
N4···H8B^viii^	2.8700	H7···H2C	2.4600
C4···Cl1^ix^	3.5458 (17)	H8A···Cl1^xii^	2.8400
C5···Cl1^ix^	3.6498 (17)	H8A···H9A	2.0700
C8···Cl3^x^	3.648 (3)	H8B···C11	2.7900
C10···C13^iii^	3.509 (3)	H8B···H11	2.3400
C10···C11^viii^	3.533 (2)	H8B···Cl3^viii^	3.0300
C11···C10^viii^	3.533 (2)	H8B···N4^viii^	2.8700
C12···Cl1^iii^	3.549 (2)	H8B···C13^viii^	2.8000
C13···C10^iii^	3.509 (3)	H8C···Cl3^x^	3.0000
C13···Cl3^i^	3.4048 (19)	H8C···C11	2.8300
C13···C14^iii^	3.491 (3)	H8C···H11	2.3900
C14···C13^iii^	3.491 (3)	H8C···H8C^xiii^	2.3600
C14···N4^iii^	3.394 (2)	H9A···H8A	2.0700
C1···H4A	2.4900	H9A···C2^xi^	2.8000
C2···H9A^xi^	2.8000	H9A···H2A^xi^	2.2800
C2···H7	2.5600	H9B···C14	2.8000
C3···H2B^xi^	2.9600	H9B···H14	2.3500
C4···H1B	2.7700	H9C···C14	2.7800
C4···H1C	2.7400	H9C···H14	2.3300
C5···H1B^vii^	3.0700	H11···C8	2.5500
C6···H1B^vii^	2.8800	H11···H8B	2.3400
C7···H2B^xi^	3.0600	H11···H8C	2.3900
C7···H2B	2.7800	H11···Cl3^viii^	2.8600
C7···H2C	2.8500	H12···Cl1^iii^	3.0400
C7···H1B^vii^	2.9700	H13···Cl3^i^	2.8100
C8···H11	2.5500	H14···Cl1	2.8700
C9···H2A^xi^	2.9400	H14···C9	2.5300
C9···H14	2.5300	H14···H9B	2.3500
C11···H8B	2.7900	H14···H9C	2.3300
C11···H8C	2.8300		
			
Cl1—Co—Cl2	113.59 (2)	N1—C2—H2C	109.00
Cl1—Co—Cl3	115.11 (2)	C3—C4—H4A	120.00
Cl1—Co—N2	104.37 (4)	C5—C4—H4A	120.00
Cl2—Co—Cl3	104.29 (2)	N2—C5—H5	118.00
Cl2—Co—N2	114.18 (4)	C4—C5—H5	118.00
Cl3—Co—N2	105.29 (4)	C7—C6—H6	118.00
C1—N1—C2	118.24 (16)	N2—C6—H6	118.00
C1—N1—C3	119.92 (15)	C6—C7—H7	120.00
C2—N1—C3	121.09 (17)	C3—C7—H7	120.00
Co—N2—C5	121.29 (11)	C11—C10—C14	115.84 (15)
Co—N2—C6	122.80 (12)	N3—C10—C11	122.27 (15)
C5—N2—C6	115.81 (14)	N3—C10—C14	121.88 (16)
C9—N3—C10	121.60 (18)	C10—C11—C12	120.02 (16)
C8—N3—C9	116.52 (19)	N4—C12—C11	121.46 (16)
C8—N3—C10	121.76 (16)	N4—C13—C14	121.57 (17)
C12—N4—C13	120.55 (16)	C10—C14—C13	120.53 (17)
C13—N4—H4	120.00	N3—C8—H8A	109.00
C12—N4—H4	120.00	N3—C8—H8B	109.00
N1—C3—C4	121.87 (15)	N3—C8—H8C	109.00
C4—C3—C7	115.53 (14)	H8A—C8—H8B	109.00
N1—C3—C7	122.61 (15)	H8A—C8—H8C	109.00
C3—C4—C5	120.32 (15)	H8B—C8—H8C	109.00
N2—C5—C4	124.10 (15)	N3—C9—H9A	109.00
N2—C6—C7	124.37 (16)	N3—C9—H9B	109.00
C3—C7—C6	119.83 (15)	N3—C9—H9C	109.00
H1A—C1—H1B	109.00	H9A—C9—H9B	109.00
H1A—C1—H1C	109.00	H9A—C9—H9C	109.00
N1—C1—H1C	109.00	H9B—C9—H9C	109.00
N1—C1—H1A	109.00	C10—C11—H11	120.00
N1—C1—H1B	109.00	C12—C11—H11	120.00
H1B—C1—H1C	109.00	N4—C12—H12	119.00
H2B—C2—H2C	109.00	C11—C12—H12	119.00
N1—C2—H2B	109.00	N4—C13—H13	119.00
N1—C2—H2A	109.00	C14—C13—H13	119.00
H2A—C2—H2B	109.00	C10—C14—H14	120.00
H2A—C2—H2C	109.00	C13—C14—H14	120.00
			
Cl1—Co—N2—C5	144.99 (11)	C9—N3—C10—C14	1.6 (3)
Cl1—Co—N2—C6	−38.86 (13)	C8—N3—C10—C11	−2.9 (3)
Cl2—Co—N2—C5	−90.41 (12)	C12—N4—C13—C14	0.5 (3)
Cl2—Co—N2—C6	85.75 (13)	C13—N4—C12—C11	−0.8 (3)
Cl3—Co—N2—C5	23.37 (13)	C7—C3—C4—C5	1.9 (2)
Cl3—Co—N2—C6	−160.48 (12)	N1—C3—C4—C5	−177.79 (15)
C1—N1—C3—C4	2.7 (2)	N1—C3—C7—C6	178.90 (16)
C1—N1—C3—C7	−176.94 (16)	C4—C3—C7—C6	−0.8 (2)
C2—N1—C3—C4	172.62 (16)	C3—C4—C5—N2	−1.1 (2)
C2—N1—C3—C7	−7.0 (3)	N2—C6—C7—C3	−1.3 (3)
Co—N2—C5—C4	175.57 (12)	N3—C10—C11—C12	−177.82 (19)
C6—N2—C5—C4	−0.8 (2)	C14—C10—C11—C12	1.8 (3)
Co—N2—C6—C7	−174.29 (13)	N3—C10—C14—C13	177.53 (18)
C5—N2—C6—C7	2.1 (2)	C11—C10—C14—C13	−2.1 (3)
C8—N3—C10—C14	177.51 (18)	C10—C11—C12—N4	−0.4 (3)
C9—N3—C10—C11	−178.8 (2)	N4—C13—C14—C10	1.0 (3)

Symmetry codes: (i) −*x*+2, −*y*, −*z*+1; (ii) *x*+1, *y*, *z*; (iii) −*x*+2, −*y*+1, −*z*+1; (iv) *x*, *y*−1, *z*; (v) *x*+1, *y*−1, *z*; (vi) −*x*+2, −*y*, −*z*+2; (vii) −*x*+1, −*y*, −*z*+2; (viii) −*x*+1, −*y*+1, −*z*+1; (ix) *x*−1, *y*, *z*; (x) *x*, *y*+1, *z*; (xi) −*x*+1, −*y*+1, −*z*+2; (xii) *x*−1, *y*+1, *z*; (xiii) −*x*+1, −*y*+2, −*z*+1.

### Hydrogen-bond geometry (Å, º)

**Table:**

*D*—H···*A*	*D*—H	H···*A*	*D*···*A*	*D*—H···*A*
N4—H4···Cl2^i^	0.8600	2.6400	3.3535 (17)	142.00
N4—H4···Cl3^i^	0.8600	2.7000	3.3279 (17)	131.00
C13—H13···Cl3^i^	0.9300	2.8100	3.4048 (19)	123.00

Symmetry code: (i) −*x*+2, −*y*, −*z*+1.
